# Track data hubs enable visualization of user-defined genome-wide annotations on the UCSC Genome Browser

**DOI:** 10.1093/bioinformatics/btt637

**Published:** 2013-11-13

**Authors:** Brian J. Raney, Timothy R. Dreszer, Galt P. Barber, Hiram Clawson, Pauline A. Fujita, Ting Wang, Ngan Nguyen, Benedict Paten, Ann S. Zweig, Donna Karolchik, W. James Kent

**Affiliations:** ^1^Center for Biomolecular Science and Engineering, School of Engineering, University of California Santa Cruz, Santa Cruz, CA 95064, USA and ^2^Department of Genetics, Center for Genome Sciences and Systems Biology, Washington University School of Medicine, St. Louis, MO 63108, USA

## Abstract

**Summary:** Track data hubs provide an efficient mechanism for visualizing remotely hosted Internet-accessible collections of genome annotations. Hub datasets can be organized, configured and fully integrated into the University of California Santa Cruz (UCSC) Genome Browser and accessed through the familiar browser interface. For the first time, individuals can use the complete browser feature set to view custom datasets without the overhead of setting up and maintaining a mirror.

**Availability and implementation:** Source code for the BigWig, BigBed and Genome Browser software is freely available for non-commercial use at http://hgdownload.cse.ucsc.edu/admin/jksrc.zip, implemented in C and supported on Linux. Binaries for the BigWig and BigBed creation and parsing utilities may be downloaded at http://hgdownload.cse.ucsc.edu/admin/exe/. Binary Alignment/Map (BAM) and Variant Call Format (VCF)/tabix utilities are available from http://samtools.sourceforge.net/ and http://vcftools.sourceforge.net/. The UCSC Genome Browser is publicly accessible at http://genome.ucsc.edu.

**Contact:** donnak@soe.ucsc.edu

## 1 INTRODUCTION

The widespread use of high-throughput sequencing technology has challenged the capabilities of genomic data visualization tools as the volume and size of genome-wide datasets outpace the capacity of existing browsing technology. In response, the University of California Santa Cruz (UCSC) has repeatedly extended its popular genome-browsing tool, the UCSC Genome Browser (Kent *et al.*, 2002; Meyer *et al.*, 2013), to offer increased visualization of remotely hosted large datasets.

In recent years, UCSC added browser support for four compressed binary indexed data formats: BigBed and BigWig (Kent *et al.*, 2010), both developed at UCSC, Binary Alignment/Map (BAM) (Li *et al.*, 2009) and Variant Call Format (VCF)/tabix (Danacek *et al.*, 2011). This allowed individuals to quickly and efficiently view and share genome-wide data hosted on their own local servers using the browser’s well-established custom track mechanism. However, the limited configuration and organization options imposed by custom tracks presented a barrier to full integration of large datasets into the browser, leading many research groups to set up mirrors to visualize their tracks in a full local instance of the browser. Mirrors pose their own drawbacks: they tend to have limited visibility and distribution within the research community and incur a local maintenance overhead.

To circumvent these limitations, UCSC has introduced support for ‘track data hubs’, Internet-accessible collections of genome annotations that can be viewed on the UCSC Genome Browser alongside native annotation tracks (Dreszer *et al.*, 2012). Track data hubs provide the power and flexibility to organize, configure and fully integrate one or more large datasets into the browser and allow efficient worldwide access to the data through the familiar Genome Browser interface. Individuals experienced in setting up Genome Browser mirrors will find that setting up a track data hub is much easier. Depending on the number and complexity of the datasets, a track data hub typically can be set up in a day or two.

Like the Distributed Annotation System (DAS) (Dowell *et al.*, 2001), track data hubs provide access to annotation over the Internet, but they differ in that the server hosting a track data hub requires only HTTP access instead of a dedicated DAS server. In contrast to the extensible mark-up language format of DAS, which may be easier for a simple application to access, track data hubs present data in the native format of the data files with a rich set of options for controlling how that data should displayed. Although this requires more complicated logic on the client end to parse and display, it offers the data contributor more options for configuring and presenting the data.

Track data hub annotations are stored at the remote site as compressed binary indexed files. When a hub track is displayed in the Genome Browser, only the relevant data needed to support the view of the current genomic region are transmitted to UCSC, rather than the entire file. The transmitted data are cached on a UCSC server to expedite future access. This on-demand transfer mechanism eliminates the need to transmit large datasets across the Internet, thereby minimizing upload time into the browser.

Hub tracks are displayed in a separate track group below the browser image and can be configured and manipulated in the same fashion as native tracks. They can be incorporated into browser sessions and custom tracks in the same manner as other tracks, and the underlying data can be viewed, manipulated and downloaded using the UCSC Table Browser (Karolchik *et al.*, 2004).

## 2 IMPLEMENTATION

Complete information about setting up a track data hub is available at http://genome.ucsc.edu/goldenPath/help/hgTrackHubHelp.html. A hub requires three components: one or more datasets formatted in a compressed binary format supported by the Genome Browser, a set of text files that specify properties for the track data hub and for each of the data tracks within it and a server with Internet access to host the data and text files. The data files underlying a track do not have to reside in the same track hub directory or server as the text files, but they must be accessible through the Internet.

The Genome Browser currently supports the compressed binary formats BigWig, BigBed, BAM and VCF/tabix, described in detail at http://genome.ucsc.edu/FAQ/FAQformat.html. The Linux binaries needed to set up a track data hub are provided by UCSC at http://hgdownload.cse.ucsc.edu/admin/exe/.

The BigWig format is ideal for viewing continuous value plot data, such as read depths from short read sequencing projects or levels of conservation observed in a multiple-species alignment. BigWig files contain lists of chromosome segments that may be displayed as a bar or line graph. Although each BigWig file contains only a single value for any given base, BigWig tracks are often combined into a ‘multiWig’ display that allows multiple BigWig files to be overlaid on the same axis.

BigBed format, which is the binary indexed version of browser extensible data format, is useful for associating a name and (optionally) a color, a score and additional user-defined data with one or more related regions on the same chromosome, such as all the exons of a gene.

BAM files, binary versions of Sequence Alignment/Map (SAM) format files, consist of alignments of DNA reads (generally short) to a reference sequence, usually a complete genome. Unlike BigWig and BigBed formats, the BAM file index is contained in a separate .bai file in the same directory and with the same root file name. 

VCF files can contain annotations of single nucleotide variants, insertions/deletions, copy number variants, structural variants or other types of genomic variation. They must be compressed and indexed using tabix (http://sourceforge.net/projects/samtools/files/tabix/). Like BAM index files, the separate tabix-formatted .tbi index file must be included in the same directory as the compressed VCF file and must have the same root file name.

In addition to the data files, each track data hub requires a directory containing a minimum of three text files: a *hub.txt* file that defines the labels used to describe the hub, a *genomes.txt* file that describes the assemblies supported by the hub and a *trackDb.txt* file that describes the data files and defines their display attributes.

The trackDb.txt file, which is based on the Genome Browser .ra format, is the most complex of the text files in the hub directory. It contains a collection of stanzas, one for each data file in a given assembly, that define the display and configuration properties for each track or group of tracks (in the case of composite or super-tracks). The Track Database Definition document (http://genome.ucsc.edu/goldenPath/help/trackDb/trackDbHub.html) provides details about how to declare the dataset display settings and values used in trackDb.txt.

Optionally, each track in the hub may also have an HTML-formatted description file that provides detailed information about the data, such as the methods used to produce and validate the data, background information, display conventions, acknowledgments and reference publications.

Once constructed, a track data hub can be imported into the Genome Browser for viewing by entering the URL of the hub.txt file on the ‘My Hubs’ tab of the track data hubs web page (http://genome.ucsc.edu/cgi-bin/hgHubConnect). The Genome Browser track data hub import utility supports Internet protocols such as http://, https://, and (less efficiently) ftp://, as well as file paths relative to the hub directory hierarchy.

A track data hub may be shared with others by providing the URL of the hub.txt file needed to load the hub. Hubs of general interest to the research community can be registered at UCSC for sharing on the Genome Browser Web site by contacting the browser technical support mailing list at genome@soe.ucsc.edu (include the URL of the hub.txt file in the message). Links to registered shared hubs may be found on the ‘Public Hubs’ tab on the Genome Browser track data hubs web page (http://genome.ucsc.edu/cgi-bin/hgHubConnect).

## 3 CONCLUSIONS

Track data hubs provide a convenient efficient mechanism for importing collections of large personal datasets into the UCSC Genome Browser for browsing, analysis and sharing with the research community. We are working with other genomics tools providers, such as Ensembl (Flicek *et al.*, 2013), to standardize the track data hub interface and add support for hub displays on other genome-browsing platforms. Future plans include extending the browser’s track search mechanism to work with hubs, expanding the flexibility of track data hub organization and configuration in the browser, providing more sample files and a wizard program to facilitate track data hub construction and adding support for more data types. Recently UCSC has also added support for assembly data hubs, which enable individuals to easily extend the Genome Browser to display genome assemblies not included in the browser database.
